# Should I Target the Blood Pressure from the Arterial Line or the Cuff? A Practical Approach for Dealing with Widely Discordant Measurements

**DOI:** 10.3390/jcm14248616

**Published:** 2025-12-05

**Authors:** Nicholas Zamith, Christopher Walker, Timothy Scully, William J. Healy, Nicola Zetola

**Affiliations:** Division of Pulmonary, Critical Care and Sleep Medicine, Augusta University, Augusta, GA 30912, USA; nzamith@augusta.edu (N.Z.); wihealy@augusta.edu (W.J.H.)

**Keywords:** hemodynamic monitoring, shock, arterial catheter, non-invasive blood pressure monitoring

## Abstract

Blood pressure (BP) monitoring is essential in managing critically ill patients in the intensive care unit (ICU), particularly for ensuring adequate end-organ perfusion in hypotensive states. Invasive arterial catheters and noninvasive oscillometric cuffs are often used together, but discrepancies between the two methods are common. These differences can arise from technical factors (e.g., transducer leveling, cuff size and placement, arterial waveform damping) as well as patient-related factors (e.g., vasoconstriction, arrhythmias, altered arterial compliance). This creates a clinical dilemma: which measurement best reflects the patient’s true perfusion pressure, and how should management be guided? This review offers a practical approach for addressing discrepancies between invasive and noninvasive BP measurements in adult hypotensive ICU patients, including those with shock requiring vasopressor support. Based on contemporary data, we propose that a difference greater than 10 mmHg in mean arterial pressure (MAP) between the two methods can serve as a pragmatic threshold to trigger structured evaluation, rather than a universal definition of clinical significance. MAP is prioritized as the key variable for assessing perfusion pressure. When a discrepancy is detected, clinicians are encouraged to integrate both measurements with clinical signs of hypoperfusion and to perform a systematic assessment of technical and physiologic contributors before deciding which value should guide treatment. We present a stepwise clinical decision-making algorithm that helps practitioners (1) recognize when a discrepancy is large enough to matter, (2) evaluate perfusion using bedside and laboratory markers, (3) identify technical or anatomic reasons for discordant readings, and (4) determine when more central arterial monitoring may be appropriate. By structuring the evaluation of discordant BP measurements, this approach aims to reduce the risk of unrecognized hypotension or overtreatment, support more consistent hemodynamic decision-making, and ultimately improve the management of critically ill, hypotensive patients.

## 1. Background

Blood pressure (BP) monitoring is essential in managing critically ill patients. As a key determinant of clinical decision-making, accurate BP measurement is vital. Clinicians must understand both clinical and technical factors that can lead to erroneous readings.

BP can be monitored invasively or non-invasively. Invasive monitoring, considered the gold standard, involves continuous BP measurement through an arterial catheter but is susceptible to technical errors. Non-invasive monitoring, typically via oscillometric cuffs, calculates BP based on arterial wall oscillations. In ICU settings, both methods are often used concurrently, particularly in hypotensive patients, to ensure accuracy and detect discrepancies.

Given the importance of ensuring end-organ perfusion, invasive and non-invasive approaches for BP monitoring are often used simultaneously to assure accuracy and provide additional information that may indicate technical problems. A clinical dilemma frequently arises when measurements do not correlate. Clinicians routinely face difficult decisions when marked differences between the two measurements arise. When these discrepancies are present, the question arises of which is closer to the “true” BP that should guide the management of the patient. In this review, we will provide a practical approach to address situations in which invasive and cuff pressures are widely discordant, focusing on hypotensive patients.

## 2. What Is a “Large Blood Pressure Discrepancy”?

A “large” BP discrepancy refers to a difference between invasive and non-invasive MAP measurements that exceeds normal physiologic variation and could influence management. MAP is prioritized as it reflects perfusion pressure. Typically, peripheral (e.g., radial) MAP closely approximates central arterial MAP within ~3 mmHg [1]. A MAP difference ≥10 mmHg is widely considered clinically significant—especially if one value crosses a critical threshold (e.g., MAP 65 mmHg) [2]. For example, an invasive arterial line MAP of 58 mmHg vs. a cuff MAP of 68 mmHg (difference 10 mmHg) would be considered a large discrepancy with potential management implications [2].

Many studies confirm the high prevalence of such discrepancies (Table S1). In a cohort of septic patients, 21% of those on vasopressors had a MAP difference ≥10 mmHg between invasive and noninvasive readings that would have changed management, compared with only 3% of patients not on vasopressors [2]. Meidert et al. reported that, in hypotensive patients, oscillometric cuff MAP averaged 13 mmHg higher than simultaneous invasive MAP, and 64% of cuff measurements failed to detect a MAP <60 mmHg that was captured by the arterial line [3]. Conversely, a large ICU database study found that, in septic patients, oscillometric MAP often underestimated invasive MAP, with a mean bias of approximately −6 mmHg and wide limits of agreement [4]. Taken together, these studies show that discrepancies of 5–15 mmHg in MAP are common, and that differences ≥10 mmHg frequently have potential management implications.

In practical terms, a “large BP discrepancy” can be defined as a difference that exceeds approximately 10 mmHg in MAP or 20 mmHg in systolic BP between two otherwise reliable measurement methods, particularly when the discrepancy would lead to different therapeutic decisions. Although this threshold is not universally validated, we propose it as a pragmatic operational benchmark supported by available data, rather than a strict physiologic cutoff. The purpose of using this benchmark is to trigger systematic evaluation rather than to define a pathologic state. Ultimately, a discrepancy is “large” if it is substantial enough to potentially compromise clinical decision-making if unrecognized—especially when one value crosses a critical threshold such as MAP 65 mmHg and the other does not.

## 3. Why Do These Discrepancies Occur? What Physiologic or Technical Factors May Be Contributing?

Discrepant readings should prompt a systematic review of both technical and physiologic contributors. Neither method of BP measurement is free from errors, and often a discrepancy between the two can be explained by understanding how each of the two works and where potential problems may arise.

### 3.1. Technical Factors

***Invasive BP monitoring:***

Invasive arterial BP monitoring involves the cannulation of a peripheral artery. The catheter is connected to fluid-filled tubing. These tubes function like a “U tube”, in which pressure applied on one side of the tube (in this case, the arterial BP) produces a shift in the fluid towards the other side. The liquid in the tube is in contact with a diaphragm that moves along with the fluid in response to the transmitted pressure waveform (hydraulic coupling); a transducer converts this movement into an electrical signal that is then processed and displayed graphically on the monitor [3]. The MAP is calculated as the area under the curve of the arterial BP waveform. To obtain accurate readings, the pressure transducer needs to be leveled with the right atrium, at the mid-axillary line at the fourth intercostal space. Sources of inaccurate invasive BP readings are related to how the system functions.

**Figure 1:**
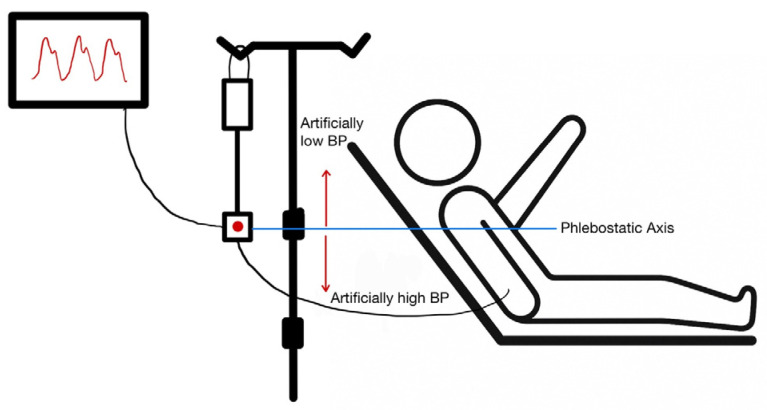
Appropriate positioning of an arterial line pressure transducer. The transducer should be aligned with the phlebostatic axis (4th intercostal space, midaxillary line). A transducer positioned below the phlebostatic axis will produce a falsely high blood pressure reading, and a transducer positioned above the phlebostatic axis will produce a falsely low blood pressure reading.

**Figure 2:**
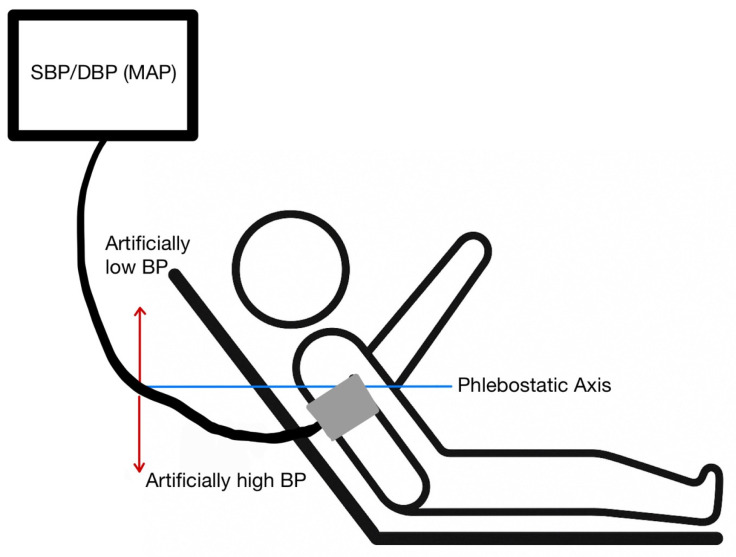
Appropriate patient positioning for a blood pressure cuff. The arm should be at the level of the phlebostatic axis. An arm raised above the phlebostatic axis will produce a falsely low blood pressure reading. An arm dropping below the phlebostatic axis will produce a falsely high blood pressure reading.

Transducer Positioning: A transducer that is not level with the right atrium can lead to erroneous readings (Figure 1 and Figure 2). A vertical difference of just 10 cm can cause a pressure difference of 7.5 mmHg [5].

- Transducer Below the Heart: Positioning the transducer lower than the phlebostatic axis results in artificially elevated pressure readings due to the added hydrostatic pressure from the fluid column [6].
- Transducer Above the Heart: Positioning the transducer higher than the phlebostatic axis leads to lower pressure readings, as the hydrostatic pressure exerted on the transducer is reduced [6].

Frequent changes in patients’ position, can result in an erroneous position of the transducer with respect to the patients’ right atrium.

**Figure 3:**
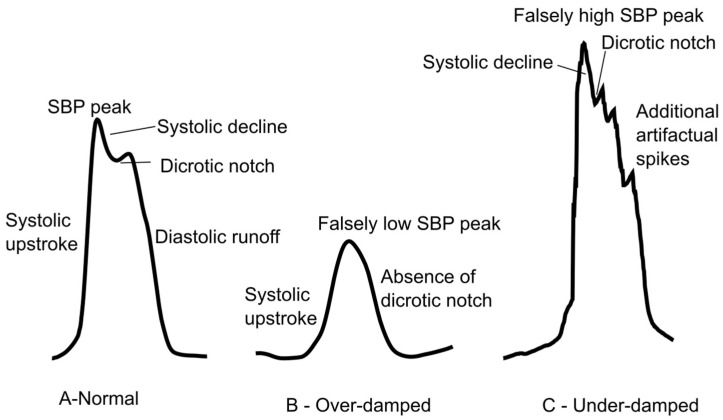
Arterial line waveform tracings. (**A**) A normal waveform demonstrating the systolic upstroke, systolic decline, dicrotic notch, and diastolic runoff. (**B**) Overdamping will cause a falsely low SBP and loss of the dicrotic notch. (**C**) Underdamping will cause a falsely high SBP with additional artifactual spikes.

Zeroing Errors: Once the transducer is well-positioned, zeroing it is essential to establish a baseline atmospheric pressure reference. Ensuring that the arterial line has been appropriately zeroed is fairly simple to do: Open the stopcock to air and verify that the monitor reads zero; if not, troubleshoot for faulty components.
Damping Issues: Over- or under-damped waveforms distort systolic and diastolic pressures. Although MAP is less affected, waveform quality impacts trustworthiness. Figure 3 shows an example of a normal, over-damped, and under-damped arterial line wave forms.
Obstructions: Air bubbles, clots, or kinks in tubing impair transmission and accuracy.

***Non-invasive BP monitoring:***

Cuff Size and Placement and Patient Positioning: Non-invasive BP measurements can be performed in a variety of different ways. In the hospital setting, this is most frequently performed with oscillometric cuffs. Oscillatory devices detect vibrations caused by blood flowing through the artery during systole and diastole, which are then transduced into electrical signals that are used by the device to calculate the BP [7]. For these measurements to be accurate, AHA guidelines recommend that patients should be seated, with back supported, legs uncrossed, and arm supported at heart level; the cuff should be on the upper arm with the cuff level with the right atrium. If patients are in the supine position, then a pillow should be used to raise the arm above the level of the heart [8]. These recommendations are often difficult to follow precisely, particularly in the ICU, and can account for discrepancies in measurements.

Additionally, cuff size should be about 20 percent greater than the diameter of the patient’s arm [9]. A cuff that is too small can lead to overestimating the BP; while an oversized cuff can underestimate it [6]. Ideally, measurements should be taken with the cuff position around the patient’s biceps (upper arm) though measurements taken from the forearm can be an alternative that correlates well [10]. Measurements from the leg should be taken in a supine position, with the cuff placed around the lower calf, ensuring that the cuff bladder encircles at least 80% of the circumference of the calf [11].

Oscillometric Device Errors: Because oscillometric BP cuffs work by measuring pulse waves, shivering or other patient movement while the measurement is being taken can lead to inaccurate readings [6]. Having the upper arm below the level of the right atrium can lead to readings that overestimate the blood pressure as well [8].

### 3.2. Anatomical and Physiologic Factors

It is important to remember that regional arterial BP gradients can cause a discrepancy between invasive and non-invasive BPs. These gradients are frequently the result of arterial obstruction caused by atherosclerosis, peripheral vascular disease, arterial embolism, or aortic dissection and they will be observed regardless of the approach used for BP measurement. In conditions with reduced arterial compliance, such as atherosclerosis and peripheral vascular disease, increased arterial resistance may affect the arterial waveform; there is an early return of reflected pressure waves which causes a late systolic pressure peak, widened pulse pressure, and disappearance of the normal diastolic runoff [6]. Non-invasive BP monitoring will also be impacted in a similar way. A study comparing non-invasive BPs using an oscillometric monitor and using a sphygmomanometer found that the oscillometric monitor, on average, overestimated the SBP by 10.9 mmHg and the DBP by 4.8 mmHg in patients determined to have stiff arteries based on carotid-femoral pulse wave velocity [12].

Because of these effects, inter-arm differences can cause one arm’s BP to be consistently higher than the other’s (i.e., large discrepancies between an invasive BP measurement taken from one arm and non-invasive BP taken from the other) [6]. In this case, both measurements represent a “true” BP for the particular extremity where the measurement was obtained but may not necessarily reflect systemic perfusion. For consistency, invasive and non-invasive measurements should ideally be taken on the same limb; otherwise, a pre-existing inter-arm gradient could falsely appear as a method discrepancy.

Discrepancy between measurements on different limbs can be present even in individuals without peripheral vascular disease, with arm BP most closely reflecting invasive measurements when compared to ankle and thigh noninvasive measurements [13].

Large gradients may also occur in certain physiologic conditions. For instance, after cardiopulmonary bypass, radial artery MAP can remain >10 mmHg lower than central aortic MAP in nearly half of patients, and in septic shock requiring high-dose norepinephrine, radial arterial pressure often underestimates central pressure by 5–6 mmHg on average, with occasional differences >15 mmHg [14,15]. Such levels of discrepancy are considered significant and potentially dangerous if unrecognized.

### 3.3. Determine the “True” Perfusion Pressure 

Which measurement reflects the true perfusion pressure? Is the lower of the two measurements acceptable, and is there clinical evidence of hypoperfusion?

When confronted with discordant blood pressure readings (e.g., an arterial line vs. a cuff pressure), the clinician’s task is to ascertain which measurement more accurately reflects the patient’s “true” perfusion pressure. Generally, the most reliable indicator of true perfusion pressure is a correctly obtained invasive arterial pressure, especially from a central arterial site [16].

However, it is not always as simple as “invasive is correct, and non-invasive is incorrect.” One must consider which artery the invasive catheter is in and the clinical context. If the arterial catheter is in a peripheral site (radial) and the patient is on high-dose vasopressors, the radial MAP may underestimate central aortic MAP [15]. In that scenario, the cuff (brachial) reading might be higher and closer to true aortic pressure. In other words, the lower reading (radial) could be falsely low due to peripheral vasoconstriction. This concept was illustrated by two studies in which femoral (central) arterial lines showed substantially higher pressures than radial lines in patients on vasopressors, meaning that the “true” perfusion pressure was actually higher than the radial-line suggested [15,17]. In such cases, simply using the lower (radial) value would underestimate organ perfusion and could lead to overzealous vasopressor use. Switching from a radial to a femoral arterial line often reveals adequate pressure and allows for de-escalation of vasopressors [17].

Conversely, if the arterial line is reliable and shows a low MAP while the cuff reads higher, clinicians should be very cautious about accepting the cuff as “true,” given that noninvasive monitors can substantially overread BP in hypotensive patients [3]. In this situation, the patient may be truly hypotensive despite a “normal” cuff pressure. The safer assumption is that the arterial line is indicating real hypoperfusion until proven otherwise. In fact, the Surviving Sepsis Campaign recommends targeting MAP ≥65 mmHg in septic shock and advocates for arterial lines largely to avoid missing such hypotension [16]. Thus, if any measurement shows MAP below a critical threshold and this is consistent with the clinical picture, the prudent approach is to address possible hypotension while evaluating the cause of the discrepancy.

The key is to integrate clinical context and patient examination when determining which value best reflects true perfusion. If the patient has signs of hypoperfusion—such as cool, mottled skin, oliguria, altered mental status, or lactic acidosis—clinicians should assume that the higher BP reading may be misleading and that the patient’s perfusion is indeed low. For example, consider an ICU patient with an arterial line MAP of 58 mmHg but a cuff MAP of 72 mmHg who is oliguric with rising lactate. The clinical picture aligns with the arterial line, and the low invasive MAP should be treated as potentially representing true inadequate perfusion pressure. Conversely, if the patient shows no signs of hypoperfusion and the only concerning element is a low reading on one device, that reading is more likely erroneous. In that scenario, it may be reasonable to provisionally consider the higher value as more reflective of true perfusion while carefully troubleshooting the discrepancy and performing frequent clinical reassessment.

Proposed Clinical Algorithm for Resolving Discordant Blood Pressure Measurements.

**Figure 4:**
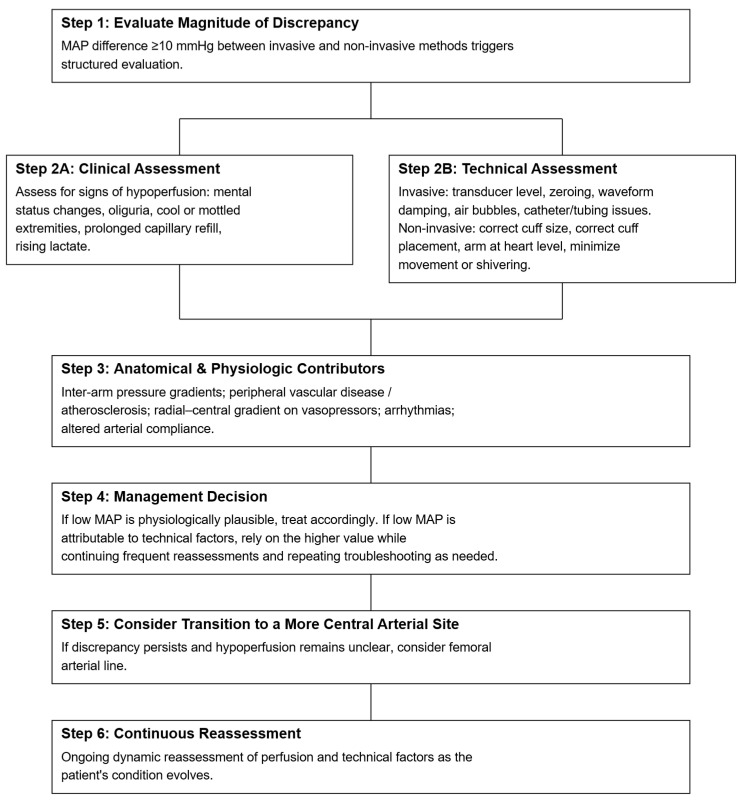
Proposed clinical algorithm for resolving discordant blood pressure measurements between invasive and non-invasive measurements. Note: This algorithm is designed specifically for situations in which simultaneously obtained invasive and non-invasive blood pressure measurements differ by ≥10 mmHg in MAP, with one measurement crossing a critical threshold (e.g., arterial line MAP 60 vs. cuff MAP 70). It is not intended to establish universal MAP goals or to replace individualized blood pressure targets. Rather, it is meant to help clinicians determine which of two discordant measurements is more physiologically plausible in the context of the patient’s clinical presentation. Importantly, “prioritizing the lower MAP” should not be interpreted as uniformly targeting a MAP of 65 mmHg; patients with vasoplegia may have hypoperfusion despite MAP ≥ 65, while those with chronic hypertension or peripheral vasoconstriction may require higher or exhibit artificially lower pressures.

Note: This algorithm is designed specifically for situations in which simultaneously obtained invasive and non-invasive blood pressure measurements differ by ≥10 mmHg in MAP, with one measurement crossing a critical threshold (e.g., arterial line MAP 60 vs. cuff MAP 70). It is not intended to establish universal MAP goals or to replace individualized blood pressure targets. Rather, it is meant to help clinicians determine which of two discordant measurements is more physiologically plausible in the context of the patient’s clinical presentation. Importantly, “prioritizing the lower MAP” should not be interpreted as uniformly targeting a MAP of 65 mmHg; patients with vasoplegia may have hypoperfusion despite MAP ≥ 65, while those with chronic hypertension or peripheral vasoconstriction may require higher or exhibit artificially lower pressures [1,2,3,15,16,17,18]. The steps that follow are therefore meant to guide interpretation of discrepant values—not to dictate fixed MAP thresholds for resuscitation (Figure 4).

Step 1: Evaluate the Magnitude of Discrepancy
  - Compare MAP from both invasive and non-invasive measurements [1,2,3,18]
    a. Significant Discrepancy: If the MAP difference between the two methods is greater than 10 mmHg, proceed with further evaluation.
    b. Non-significant Discrepancy: If the difference is less than 10 mmHg, and waveform quality and the clinical picture are reassuring, clinicians may consider both readings reliable and continue routine monitoring without further action.
Step 2: Clinical and Technical Assessment
  A. Clinical assessment: Check for indicators of hypoperfusion that suggest the patient may be under-resuscitated or inadequately perfused. Key signs include mental status changes (e.g., confusion, agitation), decreased urine output, tachycardia and tachypnea, prolonged capillary refill time (>3 s), presence of cold, clammy extremities, and rising blood lactic acid among others [16,17].
  B. Technical Assessment [5,6,7,8,9,19,20]:
    1. Rule out transducer/cuff level issues: Ensure the transducer is properly leveled at the mid-axillary line (right atrium level). A vertical difference of 10 cm can cause a 7.5 mmHg shift in BP readings. The cuff should be placed on the upper arm and at heart level. Inaccurate positioning, such as having the cuff below the heart, can falsely elevate readings.
    2. Rule out cuff size issues: Ensure that the cuff is properly sized (should encircle 80–100% of the arm). A cuff that is too small will overestimate BP, while one that is too large will underestimate it.
    3. Confirm zeroing: Ensure that the system is correctly zeroed to atmospheric pressure.
    4. Evaluated waveform (fast-flush): Check for under- or over-damping of the arterial waveform, as this can cause systolic/diastolic discrepancies. MAP remains relatively unaffected by damping errors, but correcting the damping issue can improve accuracy
    5. Inspect catheter system for obstruction: Check for air bubbles, clotting, or kinks in the catheter system tubing, which could cause inaccurate pressure transmission
Step 3: Consider Anatomical and Physiological Factors [6,11,12,13,14,15,21,22,23]
  - Evaluate for regional arterial pressure gradients: Arterial obstruction (e.g., peripheral vascular disease, atherosclerosis, embolism, or aortic dissection) can cause BP discrepancies between limbs or between invasive and non-invasive methods, even if both readings are technically accurate. Ensure that measurements are taken from the same limb and on the same side of the body. Consider repeating BP measurements using both methods in different extremities if arterial obstruction is suspected. Additionally, clinicians should consider situations in which blood pressure discrepancy between two arms would have diagnostic significance rather than being attributable to technical errors, such as in aortic dissection.
  - Consider other physiological factors: Conditions like arrhythmias, severe vasoconstriction, or altered arterial compliance can contribute to discordant BP readings. Be cautious in interpreting non-invasive measurements in patients with arrhythmias or peripheral vasoconstriction, as oscillometric devices may provide inaccurate results in these settings.
Step 4: Make management decision [3,15,16,17,24]
  - After completing the clinical and technical assessments:
    - If the low MAP appears physiologically plausible and is accompanied by signs of hypoperfusion that are not explained by technical factors, treat accordingly as true circulatory compromise.
    - If the low MAP is more likely attributable to technical issues and there is no clinical evidence of hypoperfusion, it may be reasonable to provisionally rely on the higher value while continuing frequent patient reassessments and repeating troubleshooting as needed.
Step 5: Consider transitioning to a more central arterial site (e.g., femoral)
  - Radial MAP may be spuriously low due to severe vasoconstriction (e.g., high-dose vasopressors). In this circumstance, clinicians may consider the higher brachial cuff as potentially more reflective of true central perfusion pressure. If there is evidence of hypoperfusion, a transition to a femoral arterial line should be considered in order to distinguish spuriously low radial pressure from true central hypotension [14,15,17].
Step 6: Continuous Monitoring and Reassessment
  - Regularly reassess both invasive and noninvasive BP measurements, along with clinical markers of perfusion, and repeat troubleshooting as needed—especially if the patient’s status changes or discrepancies recur. Because averaging two discrepant MAP values can mask true hypotension and delay recognition of arterial line malfunction, averaging is not recommended as a decision-making strategy. Persistent unexplained discordance should prompt reconsideration of the monitoring strategy (e.g., resisting the arterial line, obtaining a more central arterial line, or using complementary hemodynamic assessments).

## 4. Conclusions

Blood pressure monitoring is a vital component of caring for critically ill patients, yet simultaneous invasive and noninvasive measurements frequently disagree in ways that can obscure the patient’s true perfusion pressure. This review proposes a practical, stepwise algorithm to structure the evaluation and management of widely discordant BP measurements in hypotensive adult ICU patients. The framework begins with defining a pragmatic threshold for a “large” discrepancy (≥10 mmHg in MAP), then guides clinicians through clinical and technical checks, consideration of anatomic and physiologic contributors, and decisions about when to transition to more central arterial monitoring.

By integrating invasive and noninvasive values with global indicators of perfusion, the algorithm is intended to reduce the risk of unrecognized hypotension, avoid unnecessary escalation of vasopressor therapy, and promote more consistent bedside decision-making. In practice, this structured approach could be incorporated into ICU protocols, educational curricula for trainees, and electronic decision support tools to standardize the evaluation of discordant blood pressure readings. Future work should focus on prospectively validating the algorithm, quantifying its impact on workflow and patient outcomes, and refining thresholds and decision points as additional evidence emerges.

## Supplementary Materials

The following supporting information can be downloaded at: https://www.mdpi.com/article/10.3390/jcm14248616/s1, Table S1: Studies Comparing Blood Pressure Measurements across Various Approaches, Sites, and Technical Errors.
