# Supplementary material for: Should I Target the Blood Pressure from the Arterial Line or the Cuff? A Practical Approach for Dealing with Widely Discordant Measurements
Source: J Clin Med. 2025 Dec 5;14(24):8616. doi: 10.3390/jcm14248616 (PMC12733910; doi:10.3390/jcm14248616)
Supplement: Supplementary file 1 [file jcm-14-08616-s001.zip › jcm-4000257-supplementary.pdf]

Studies Comparing Blood Pressure Measurements across Various Approaches, Sites, and Technical Errors

Numerous studies have examined the magnitude of differences between invasive and non-invasive BP measurements across different populations and clinical settings. Table S1 summarizes key findings from large observational studies and meta-analyses, highlighting the variability in systolic, diastolic, and mean pressures. These data underscore the lack of fixed thresholds for clinical relevance and support our proposed cutoffs for identifying meaningful discrepancies.

Reference	Year	Number of Patients	Study Design	Main Results	Conclusions
**Kaufmann et al., J Crit Care [4]**	2020	736	Post hoc analysis of a prospective study comparing NIBP vs invasive BP in ICU patients	Minimal average SBP difference (0.8 mmHg), wide limits of agreement (~±15.7 mmHg)	Wide variability; NIBP not consistently over/underestimated; invasive preferred for precision
**Meidert et al., J Clin Monit Comput [3]**	2021	30	Prospective observational study in ED shock patients; 75 paired measurements	MAP by cuff overestimated invasive MAP by 13 ± 15 mmHg; 64% of cuff MAPs missed hypotension	NIBP unreliable in hypotension; invasive measurement recommended in shock
**Liu et al., Blood Pressure [21]**	2016	742	Retrospective MIMIC-II database study comparing NIBP vs invasive BP across age groups	Small SBP differences by age; variability increases with age (elderly ~3 mmHg mean difference)	Agreement worsens with age; NIBP slightly overestimates SBP in elderly
**Jiang et al., J Anesthesia [18]**	2024	6060	Retrospective MIMIC-IV database study in sepsis patients; 96,000+ BP pairs	Mean MAP by cuff was ~6 mmHg lower than invasive MAP; SBP bias near zero	NIBP and IBP not interchangeable; invasive preferred in sepsis for MAP accuracy
**Cheng et al., Int J Cardiol [22]**	2013	857 (meta-analysis)	Systematic review of applanation tonometry vs invasive aortic pressure	Cuff SBP overestimated central SBP by ~8–10 mmHg; MAP bias ~5 mmHg	Cuff BP differs significantly from central pressure; tonometry more accurate when calibrated
**Siaron et al., Sci Rep [22]**	2020	80	Cross-sectional study of BP at 4 limb sites using NIBP and invasive BP where available	Median inter-site SBP range: 10 mmHg; MAP range: 6 mmHg; all p < 0.05	BP readings vary by site; standardization of site is critical for accuracy

**Yüksel et al., Florence Nightingale J Nurs. [19]**	2020	100	Prospective crossover trial in obese patients using standard vs large cuff	Too-small cuff overestimated SBP by ~20.8 mmHg; DBP ~10.2 mmHg; $p < 0.001$	Improper cuff size inflates BP readings significantly; large cuff needed in obesity
**Saherwala et al., Neurocrit Care [25]**	2018	70	Prospective study in neuro-ICU patients on vasoactive meds comparing NIBP vs intra-arterial BP	Cuff SBP ~4 mmHg lower than IAP on average; >20 mmHg discrepancy in ~25% cases	Cuff and invasive BP often differ under vasoactives; invasive preferred for accuracy
**Kho et al., Obstet Med [20]**	2009	219	Prospective study comparing standard vs large cuff in pregnant women	Standard cuff overestimated SBP by ~5–7 mmHg in women with large arm circumference	Correct cuff sizing essential in pregnancy to avoid BP overestimation
**Wiecek et al., Am J Cardiol [27]**	1990	15	Observational study comparing cuff vs arterial BP during rest and exercise	Cuff SBP ~19 mmHg lower than invasive during exercise; not specific to arm position	Cuff underestimates BP during exertion; not a study on arm positioning
**Lehman et al., Crit Care Med [28]**	2013	Not specified	Conceptual/mechanistic discussion on arterial line zeroing and transducer height	10 cm transducer misplacement causes ~7.5 mmHg BP error	Zeroing at right atrial level is essential for accurate arterial BP readings
**Kim et al., Anesth Analg [29]**	2014	60	Simulation/observational study on arterial transducer misplacement	Transducer offset by 10 cm caused ~7.5 mmHg MAP error	Proper transducer leveling critical to avoid false BP readings
**Seidlerova et. Al, BMC Cardiovascular Disorders [30]**	2019	85	Prospective study comparing agreement between NIBP vs intra-arterial BP in patient with cardiogenic shock	In setting of shock, the accuracy of non-invasive MAPs was very low with a concordance correlation coefficient of 0.36	NIBP and IBP not interchangeable; invasive preferred in cardiogenic shock for MAP accuracy
**Lakhal et al., Br J. Anaesth. [21]**	2015	135	Prospective study comparing NIBP vs intra-arterial BP in patient with arrhythmia vs in patients with regular rhythm	Agreement between NIBP vs intra-arterial BP was similar to that observed in 136 patients with regular rhythm	Arrhythmia did not impact automated cuff reading as previously assumed

**Kaur et al., Blood Press Monit [32]**	2019	36	Comparative analytical study comparing NIBP vs intra-arterial BP in patient with arrhythmia vs in critically ill patients receiving inotropes	MAP not evaluated. A noted difference was clinically acceptable in 54.2% of SBP measurements and 74.1% DBP measurements	Observed difference in measurements increased with increasing inotrope used, however unclear how clinical decision-making would change, as MAP was not evaluated
**Wax, et al., Anesthesiology [33]**	2011	15,310	Retrospective study comparing NIBP vs intra-arterial BP in patients undergoing anesthesia for non-cardiac cases NIBP vs intra-arterial BP in patient	NIBP measurements overestimated the BP as patients became more hypotensive and underestimated the BP as patients became more hypertensive. This trend was noted for systolic, diastolic, and MAP measurements. The crossover points were: 111 mmHg for SBP, 80 mmHg for DBP, and 95 mmHg for MAP	NIBP becomes less reliable at physiologic extremes of blood pressure (both hypo- and hypertension)
**Lakhal, et al. Crit Care Med [13]**	2012	150	Prospective observational study comparing MAP taken through NIBP at three sites (arm/thigh/ankle) vs intra-arterial MAP	Arm NIBP measurements were the most reliable (mean bias of 3.4 ± 5.0 mmHg and limits of agreement between -6.3 and 13.1 mmHg).<br><br>Ankle and thigh NIBP measurements showed larger discrepancies (mean biases of 3.1 ± 7.7 mmHg and 5.7 ± 6.8 mmHg, and limits of agreement of -12.1 to 18.3 mmHg and -7.7 to 19.2 mmHg, respectively)	Generally, arm noninvasive MAP readings most closely reflect invasive measurements compared to other locations.
